# Bringing the commercial determinants of health out of the shadows: a review of how the commercial determinants are represented in conceptual frameworks

**DOI:** 10.1093/eurpub/ckz197

**Published:** 2020-01-18

**Authors:** Nason Maani, Jeff Collin, Sharon Friel, Anna B Gilmore, Jim McCambridge, Lindsay Robertson, Mark P Petticrew

**Affiliations:** 1 London School of Hygiene and Tropical Medicine, London, UK; 2 School of Public Health, Boston University, Boston, MA, USA; 3 Global Health Policy Unit, School of Social and Political Science, University of Edinburgh, Edinburgh, UK; 4 SPECTRUM Consortium, UK; 5 School of Regulation and Global Governance (RegNet), College of Asia and the Pacific, The Australian National University, Canberra, ACT, Australia; 6 Tobacco Control Research Group, Department for Health, University of Bath, Bath, UK; 7 Department of Health Sciences, University of York, Heslington, York, UK

## Abstract

**Background:**

The term ‘commercial determinants of health’ (CDOH) is increasingly focussing attention upon the role of tobacco, alcohol and food and beverage companies and others—as important drivers of non-communicable diseases (NCDs). However, the CDOH do not seem to be clearly represented in the most common social determinants of health (SDOH) frameworks. We review a wide range of existing frameworks of the determinants of health to determine whether and how commercial determinants are incorporated into current SDOH thinking.

**Methods:**

We searched for papers and non-academic reports published in English since 2000 describing influences on population health outcomes. We included documents with a formal conceptual framework or diagram, showing the integration of the different determinants.

**Results:**

Forty-eight framework documents were identified. Only one explicitly included the CDOH in a conceptual diagram. Ten papers discussed the commercial determinants in some form in the text only and fourteen described negative impacts of commercial determinants in the text. Twelve discussed positive roles for the private sector in producing harmful commodities. Overall, descriptions of commercial determinants are frequently understated, not made explicit, or simply missing. The role of commercial actors as vectors of NCDs is largely absent or invisible in many of the most influential conceptual diagrams.

**Conclusions:**

Our current public health models may risk framing public health problems and solutions in ways that obscure the role that the private sector, in particular large transnational companies, play in shaping the broader environment and individual behaviours, and thus population health outcomes.

## Introduction

Over the last few decades, the complex interactions between individuals, communities and their environments have been a focus of public health practice and research, and to a lesser extent policy-making. These interactions are conceptualized through frameworks, often prominently involving graphical schematic diagrams illustrating the different determinants of health and how they inter-relate. The most well-known of these is probably Whitehead and Dalhgren’s rainbow.^1^ The value of such frameworks has been made clear in a report for the World Health Organization (WHO) Regional Office for Europe, in which Whitehead and Dalhgren themselves noted that: ‘To be effective in tackling social inequities in health, policy-makers and practitioners need a sound understanding of the current evidence about the key determinants and ways in which health systems can confront them in different country contexts’.^1^

As well as being an aid to policy and practice, such frameworks also serve as a research tool, helping to guide research, e.g. by helping the development of hypotheses about the relative influence of these determinants and their interactions. They also help to shape consideration of policy options, and to identify leverage points for new interventions, as well as facilitating communication between academic and non-academic stakeholders.^2^

However, although such frameworks generally aim to represent the wider determinants of population health, the commercial or private sector often does not appear to be explicitly or prominently included. This is an important omission because there are increasing calls for greater recognition of the negative impacts on health arising from commercial activities.^3–5^ The term commercial determinants of health (CDOH) has being used to focus attention upon this concept,^3–5^ and arises from a recognition that tobacco, alcohol, and food and beverage companies and other harmful commodity producers—through both their market and non-market activities—are important and often-overlooked drivers of non-communicable diseases (NCDs) in both high-, low- and middle-income countries.^5^ This is reflected in the Vienna Declaration on Public Health, a 30-year renewal of the Ottawa Charter, which notes that:

it is essential to recognize the growing role of diverse non-state actors, and especially the importance of distinguishing those that pursue public interest objectives from those that pursue commercial interests, paying particular attention to the difficulties that can arise with activities promoted as corporate social responsibility.^6^^,^^7^

Such statements reflect the extent to which commercial actors, often with resources far in excess of national or non-governmental counterparts, are seen to be able to influence population health and wellbeing at the supranational, national, community and individual levels.

The evidence regarding the nature and extent of influence of commercial influences on population health is now sufficiently well established that it would seem odd not to include commercial actors among the main determinants of health. This article aimed to review a wide range of existing frameworks of the determinants of health to determine whether and how commercial determinants appear to be incorporated into current thinking. The study objectives were (i) to identify the most well-known and most cited frameworks, and (ii) to analyze whether and how commercial influences and the activities of commercial actors were reflected in conceptual frameworks and diagrams.

The overall aim was to identify the most salient, widely known, and widely cited frameworks, primarily based on database searches, and searches of their reference lists, and supplemented with our own knowledge of the field, as an entry point for discussion of how thinking and practice around the CDOH can be developed further.

## Methods

### Search strategy

We initially conducted a scoping search on Medline for ‘social determinants’+ ‘framework’, from 1 January 2000 to 6 September 2017, resulting in 615 abstracts in total. We used Google Scholar and searches of the bibliographies of retrieved papers to identify any relevant non-academic reports (e.g. policy documents) and frameworks from pre-2000. We supplemented the list with the authors’ own knowledge of existing frameworks, if they met the inclusion criteria.

### Inclusion criteria

We included in our final analysis any documents if they included some form of formal conceptual framework graphical figure or diagram, showing the integration of the different determinants. We set out to identify framework documents that explicitly set out the relationship between population health outcomes and/or health inequalities, and their contributing factors (usually at different levels from the individual up to the societal level), either to explain the aetiology of conditions and/or to identify intervention entry points.

### Exclusion criteria

We excluded general discussion documents that did not contribute to overarching frameworks—on the grounds that it is the conceptual frameworks that are likely to be most influential [such as the Commission on the Social Determinants of Health (CSDH) framework].^8^ For the same reason, we excluded life-course models, as these tend to be epidemiologically based models of exposures, rather than analyses of wider influences on individuals and contexts. We did not include frameworks focussed on a single condition subpopulation (unless it appeared to be a broadly applicable framework).

### Coding

We sorted the retrieved papers in date order and coded them based on whether or not they contained reference to the concept of commercial determinants in a framework, or just in the text. We also coded instances where the private sector was discussed positively in the context of a framework or text, and whether the framework papers included examples of either harmful or positive influence.

## Results

### Inclusion of commercial determinants of health

We identified 48 of the most common conceptual frameworks for the social determinants (see Supplementary table S1; for full list of references to frameworks, see Supplementary material). Of these frameworks, only one explicitly included commercial actors, in the context of the food industry and in the form of a table.^9^ Ten papers discussed the concept of commercial determinants in some form in the text, and fourteen gave examples of corporate influence or negative impacts. Twelve papers discussed the concept of a positive, health enhancing role for the private sector (presented as either text and/or a figure). Exclusions were typically generally review papers, or opinion papers which mentioned other frameworks, without proposing or developing conceptual social determinants of health (SDOH) frameworks themselves.

### Health impact of commercial determinants

There was little or no explicit recognition of the scope for analyzing the health impacts, or roles of, commercial actors in many of these frameworks (Supplementary table S1), which often focus on broader structural determinants and include generic terms such as ‘economic’ determinants. Such generic constructs as socioeconomic, material, work or occupational factors are, of course, highly relevant to consideration of social determinants, though are largely distinct from commercial determinants in the form of the consequences of the business activities of private sector actors, the core subject matter of this paper.

Even when economic activity and trade was included in a conceptual diagram, the focus appeared most often to be on the general macroeconomic environment, and not on the ways in which specific commercial actors such as transnational corporations can impact trade agreements, drive consumer demand, provide employment, shape working conditions, obtain partnerships with public sector agencies, deliver healthcare services or influence policies and regulations.^10^^,^^11^ On the other hand, some frameworks did not even include economic determinants (e.g. the Meikirch model,^12^Supplementary table S1).

### References to harmful products

In some cases, even where commercial determinants were included in the text indirectly (e.g. via foci on tobacco and alcohol) they appear to be presented as ‘lifestyle factors’ without including the commercial actors which drive their consumption.^13^ (e.g. WHO’s Health in All Policies comprehensive Framework^14^; Supplementary table S1). The burden of diseases and injuries consequent on use of many unhealthy commodities is strongly influenced by factors such as availability, pricing and advertising as part of a comprehensive marketing mix, all of which involve decision-making by private sector actors to further business interests. These were generally found to be absent from frameworks which include a focus on individual ‘lifestyles’. In some cases, availability was mentioned in the text (e.g. the recent Health Foundation report,^15^) but marketing—an important commercial determinant—was, across all frameworks, very rarely mentioned. The US Centre for Disease Control and Prevention’s (CDC) use of a social ecologic model for violence prevention^16^ is an example of the omission of a key commercial determinant, as the CDC framework does not include firearms or the firearm industry, and alcohol or the alcohol industry, despite the roles both play in violence in the USA.^17–19^ Another such example is the Framework for Patterning of Women’s Health, which does not mention determinants such as marketing, although women may be disproportionately targeted by some forms of harmful product marketing, or be at a disproportionate risk from some products (such as alcohol).^20^

Although commercial determinants generally do not appear in the conceptual diagrams, some of the supporting text does describe them at length. There were ten examples of this (Supplementary table S1). For example Dahlgren and Whitehead’s ‘Levelling Up Part 2’ discusses globalization and the influence of major financial players, as well as specific references to examples of commercial determinants driving inequalities.^21^ Similarly, in a framework for diet-related NCDs, Libman et al.^9^ explicitly reference the food industry’s influence, from a micro (community) to a macro (global) context, and describes the difficulties that arise due to conflicts of interest between population health and profits. In the context of health and globalization, Labonté and Torgerson also discuss corporate power and influence as part of global capitalism ‘is substantially shifting power away from public governing bodies and towards private economic organisations, the power of which is defined by national and supranational structures of property rights’.^22^ In the same context, Huynen etal.^23^ note both the role of public–private partnerships and the role of commercial entities in promoting NCDs: ‘Although the major chronic diseases are not transmissible via an infectious agent, the behaviours that predispose to these diseases can be communicated by advertising, product marketing and social interactions’.^23^

The recent report from The Health Foundation and the Institute of Health Equity on the role of charities in addressing the SDOH ,^15^ does not directly identify a construct such as commercial determinants, though does refer to fast food shops and a lack of available healthy produce as an environmental determinant entitled ‘Our surroundings’.^15^ Similarly, this report identifies betting shops and payday loans as increasing the risk of financial difficulty in deprived areas. Finally, a chapter on ‘Market Responsibility’ in the CSDH final report^8^ also recommends that robust public health leadership is needed to control the circulation of health-damaging commodities such as tobacco and alcohol, and notes that processed foods and alcohol are prime candidates for stronger global, regional, and national regulatory controls. It also highlights the importance of global governance mechanisms such as the Framework Convention on Tobacco Control. One of its recommendations is to: ‘Reinforce the primary role of the state in the provision of basic services essential to health (such as water/sanitation) and the regulation of goods and services with a major impact on health (such as tobacco, alcohol, and food)’.

## Discussion

It is clear that consideration of commercial actors is frequently understated, not made explicit, or simply missing in many of the most influential conceptual frameworks addressing the SDOH, with few exceptions (e.g. Libman et al.^9^ include reference to the food industry in the context of diet-related NCD). This is particularly notable in frameworks where corporate influence could potentially have a major influence—e.g. in a framework concerning childhood tooth decay that does not include the role of the marketing of sugar products.^24^ This invisibility of the commercial determinants is highly problematic, because the absence from CDOH from many of these frameworks may obscure commercial sector responsibility for, and contributions to, health inequalities and population harms, and unhelpfully deflect attention to other social determinants. The unintended consequences may be that policymakers, practitioners and researchers will be led to misdirect their attention to the role of social policies as the appropriate remedies, whilst commercial causes of public health problems are ignored or obscured, thus omitting consideration of the need for closer regulation of harmful commodities and their producers. We are not optimistic that there is any significant increase over time in the inclusion of commercial determinants in these frameworks, though there are too few examples to be sure.

Within the frameworks we examined, public health most consistently frames public health problems as being caused by, and remedied by social policies—housing, education, employment—and changes in individual behaviours as opposed to the wider environment. Although the text of documents (like the CDOH Closing the Gap report) sometimes describes commercial determinants, this may be insufficient. This is because the conceptual diagrams (as opposed to their textual descriptions) are important communication tools, which contribute to shaping how people (like policymakers) understand problems and frame their solutions.^25^ Moreover the commercial determinants that are included in these frameworks are represented in a wide range of specific and non-specific ways—including specific commodities (e.g. alcohol, tobacco, sugar), but also as broader commercial factors such as ‘trade’ and ‘marketing’; and these are often in turn mentioned as general environmental ‘influences’, rather than as being driven by commercial actors.

### Possible solutions: putting commercial influences back in the picture

Buse et al. say that ‘…the need for a global collective response and policy coherence across sectors to effectively hold the commercial food, beverage, alcohol and tobacco industries (at least partially) accountable for public health outcomes is clear, the question of ‘how’ requires further consideration’.

Some existing frameworks do offer examples of how CDOH may be better incorporated and operationalized. For example the WHO’s 2006 report on ‘Health in All Policies’ includes one of the most thorough considerations of corporate entities of those we examined, describing the relationships between globalization, the interests of the private sector, supranational arbitration and health policy, using examples such as the pitfalls for health policy associated with ‘Better Regulation’ processes, conflicts of interest in the areas of food production, weak voluntary agreements to combat obesity, and marketing of unhealthy products to children. It also includes examples of successful initiatives to prevent undue corporate influence, such as tobacco directives (WHO, 2006). Similarly, the PHE ‘Wider Determinants of Health’ tool includes indicators of the commercial determinants—such as ‘Density of Fast Food Outlets’, and licenced alcohol outlets (see https://fingertips.phe.org.uk/profile/wider-determinants).

These findings suggest that the ‘how’ could involve developing a more explicit and systematic focus on commercial determinants—not just in the text, but also in the conceptual diagrams. There may be several ways to do this. It can be done, e.g. by strengthening existing SDOH frameworks to explicitly include the commercial determinants. It can also be done by developing a standalone model of the commercial determinants (as called for by Kickbusch, Stuckler and others).^3^^,^^4^^,^^26^ Indeed, a growing literature has attempted to consider the ways in which corporate actors may influence health.^27–29^ This includes, e.g. the framework proposed by Mialon etal.^30^ for monitoring and analyzing the political activity of the food industry but clearly this remains largely disconnected from other social determinants frameworks. This may reflect an unhelpful separation of the relevant literatures, that if integrated might offer a clearer and more complete roadmap to improving population health.

The further development of CDOH within existing social determinants frameworks may serve to highlight their importance; will mitigate concerns about generating multiple and competing agendas; will highlight the potential for regulating corporate conduct as identifiable (and perhaps more politically feasible) sites for policy intervention; and will better integrate NCD prevention policies with the research and policy agendas of actors concerns to address wider health and social inequalities. It will also be important to reflect further on the respective strengths and weaknesses of seeking to develop standalone frameworks vs. (or as well as) integrating CDOH into existing wider social determinants and health equity work. The development of a new standalone model of the CDOH may however be essential in order to give the field the necessary impetus, and to help develop the scientific and other infrastructure to develop it further.

Arguably, the underlying need, however, to develop innovations in concepts in this area, implies some movement towards integration. It may also be preferable for any updating of existing frameworks and any development of new ones to be undertaken in parallel. Some frameworks are highly targeted to specific policy or other needs, and one size may not fit all. What is also important is that as new frameworks continue to emerge, they should be scrutinized to ensure that they adequately represent the full range of commercial determinants (including, e.g. gambling and extractive industries), and do not simply perpetuate their invisibility, which is convenient for harmful industries while being harmful to public health. Note too that our review is not a systematic review, though we do not believe that our findings would have been significantly different if it had been (e.g. with more comprehensive searches), as we identified and included all the major SDOH frameworks.

## Conclusions

Public health urgently requires a rapid evolution of existing SDOH frameworks and thinking in order to extend the core concepts so that they more fully consider the CDOH.^31^ It also requires decision-making by policy actors to hold powerful commercial actors to account for their actions.^32^^,^^33^ In the absence of this, our current public health frameworks may risk framing public health problems and solutions in ways that inadvertently obscure the role that the private sector, in particular large transnational companies, play in shaping the broader environment individual behaviours, and population health outcomes.

The ever-strengthening interest in CDOH therefore requires strengthened conceptual tools. A recent Lancet editorial ended with a call to arms on the CDOH: ‘It is time for a conscious attack on commercial interests and a radical rethinking of the dominant economic and political models that have too little interest in equity or social justice’.^34^

This attack now needs to address itself to the conceptual models we use to shape our definitions of the problem, its causes, our evidence-gathering, and our proposed solutions.

## Funding

M.P.P., A.B.G., J.C. and S.F. are co-investigators in the SPECTRUM consortium which is funded by the UK Prevention Research Partnership (UKPRP), a consortium of UK funders [UKRI Research Councils: Medical Research Council (MRC), Engineering and Physical Sciences Research Council (EPSRC), Economic and Social Research Council (ESRC) and Natural Environment Research Council (NERC); Charities: British Heart Foundation, Cancer Research UK, Wellcome and The Health Foundation; Government: Scottish Government Chief Scientist Office, Health and Care Research Wales, National Institute of Health Research (NIHR) and Public Health Agency (NI)]. N.M.H. is supported via a Harkness Fellowship, funded by the Commonwealth Fund. The views presented here are those of the authors and should not be attributed to the above funding organisations, their directors, officers or staff. The open access fees for this paper were paid by the Wellcome Trust for Investigator Award 200321/Z/15/Z to J.M. J.C., S.F., A.G., L.R. and M.P. are members of the SPECTRUM Consortium, which is funded by the UK Prevention Research Partnership, an initiative funded by UK Research and Innovation Councils, the Department of Health and Social Care (England) and the UK devolved administrations, and leading health research charities.

*Conflicts of interest*: None declared.

**Key points**

- Conceptual frameworks for the ‘social’ determinants of health have frequently been used to communicate the complex interactions between individuals, communities and their environments.
- However the role of the ‘commercial or private sector’ (AKA the commercial determinants of health)—does not appear to be explicitly or prominently included in many of the main social determinants frameworks.
- This study reviewed a wide range of commonly cited and influential social determinants frameworks. It found that descriptions of commercial determinants are frequently understated, not made explicit, or are very often simply missing.
- Our current public health models—and the policies which they inform—therefore risk framing public health problems and solutions in ways that inadvertently obscure the role that the private sector, in particular large transnational companies, play in shaping population health outcomes.

## Supplementary Material

ckz197_Supplementary_Data
