# Supplementary material for: Bringing the commercial determinants of health out of the shadows: a review of how the commercial determinants are represented in conceptual frameworks
Source: Eur J Public Health. 2020 Jan 18;30(4):660–4. doi: 10.1093/eurpub/ckz197 (PMC7445044; doi:10.1093/eurpub/ckz197)
Supplement: ckz197_Supplementary_Data [file ckz197_supplementary_data.zip › ejph-2019-07-om-0571-File002.docx]

**Supplementary Table 1: 48 conceptual frameworks for the social determinants of health**

| **Framework** | **Concept of CDOH** | | **Positive role for commercial sector** | **Examples of negative commercial influence** | **Examples of positive commercial role** | **No mention of CDOH** |
| --- | --- | --- | --- | --- | --- | --- |
|  | **Framework diagram** | **Text** |  |  |  |  |
| A new perspective on the health of Canadians(34) | **-** | **+** | **-** | **-** | **-** | **-** |
| Inequalities in health: Reports of a research working group(35) | **-** | **-** | **-** | **-** | **-** | **-** |
| Producing health, consuming health care(36) | **-** | **+** | **-** | **+** | **-** | **-** |
| Population Health Promotion: An Integrated Model of Population Health and Health Promotion(37) | **-** | **-** | **-** | **-** | **-** | **+** |
| From social integration to health: Durkheim in the new millennium(38) | **-** | **-** | **-** | **+** | **-** | **-** |
| Basic concepts in population health and health care(39) | **-** | **-** | **-** | **-** | **-** | **+** |
| The Social-Ecological Model: A Framework for Violence Prevention(40) | **-** | **-** | **-** | **-** | **-** | **+** |
| Gender equity and socioeconomic inequality: a framework for the patterning of women’s health(20) | **-** | **-** | **-** | **-** | **-** | **+** |
| Social determinants of health: implications for environmental health promotion(41) | **-** | **-** | **-** | **-** | **-** | **+** |
| The health impacts of globalisation: a conceptual framework(23) | **-** | **+** | **-** | **+** | **-** | **-** |
| European strategies for tackling social inequities in health: Levelling up Part 2(21) | **-** | **+** | **+** | **+** | **-** | **-** |
| Health in All Policies: Prospects and Potentials(42) | **-** | **+** | **+** | **+** | **+** | **-** |
| Measuring population health: A review of  indicators(43) | **-** | **-** | **-** | **-** | **-** | **+** |
| Social Determinants of Health(44) | **-** | **-** | **-** | **-** | **-** | **+** |
| Levelling up part 1: Concepts and principles for tackling social inequities in health(45) | **-** | **-** | **-** | **-** | **-** | **+** |
| A health map for the local human habitat(13) | **-** | **-** | **-** | **-** | **-** | **-** |
| The links between agriculture & health: an intersectoral opportunity to improve the health and livelihoods of the poor(11) | **-** | **-** | **-** | **-** | **-** | **+** |
| Influences on children's oral health: a conceptual model(24) | **-** | **-** | **-** | **-** | **-** | **+** |
| A typology of actions to tackle social inequalities in health(46) | **-** | **-** | **-** | **+** | **-** | **-** |
| Social determinants of Métis health(47) | **-** | **-** | **-** | **-** | **-** | **+** |
| The Social Determinants of Cancer: A Challenge for Transdisciplinary Science(48) | **-** | **+** | **-** | **-** | **-** | **+** |
| Health as freedom: addressing social determinants of global health inequities through human right to development(49) | **-** | **-** | **-** | **-** | **-** | **+** |
| Social Determinants of Health (Second Edition ed.)(50) | **-** | **-** | **-** | **-** | **-** | **+** |
| Tackling the wider social determinants of health and health inequalities: evidence from systematic reviews(51) | **-** | **-** | **-** | **-** | **-** | **+** |
| Rethinking the 'global' in global health: a dialectic approach(52) | **-** | **-** | **-** | **-** | **-** | **+** |
| The Canadian Facts(53) | **-** | **-** | **-** | **+** | **-** | **-** |
| Conceptual Framework for Action on the Social Determinants of Health(54) | **-** | **-** | **-** | **-** | **-** | **+** |
| The Social Determinants of Health: Coming of Age(55) | **-** | **-** | **-** | **-** | **-** | **+** |
| U.S. Health in International Perspective: Shorter Lives, Poorer Health(56) | **-** | **-** | **-** | **-** | **-** | **+** |
| Health in global context; beyond the social determinants of health?(57) | **-** | **-** | **-** | **-** | **-** | **+** |
| Total Environmental Assessment Model for Early Childhood Development(58) | **-** | **-** | **-** | **+** | **+** | **-** |
| Health in all policies: Helsinki statement. Framework for country action(59) | **-** | **+** | **+** | **+** | **+** | **-** |
| Evidence review: Addressing the social determinants of inequities in mental wellbeing of children and adolescents (60) | **-** | **-** | **-** | **-** | **-** | **+** |
| VicHealth Framework for Health Equity (61) | **-** | **-** | **-** | **-** | **-** | **+** |
| Evidence review: Settings for addressing the social determinants of health inequities (62) | **-** | **-** | **+** | **-** | **-** | **+** |
| Evidence review: The social determinants of inequities in alcohol consumption and alcohol-related health outcomes (63) | **-** | **-** | **+** | **-** | **-** | **+** |
| Evidence review: Early childhood development and the social determinants of health inequities (64) | **-** | **-** | **-** | **+** | **-** | **-** |
| Evidence review: Addressing the social determinants of inequities in healthy eating (60) | **-** | **+** | **+** | **+** | **-** | **-** |
| Evidence review: Addressing the social determinants of inequities in physical activity and related health outcomes (65) | **-** | **-** | **-** | **-** | **-** | **+** |
| Evidence review: Social innovation for health equity promotion (66) | **-** | **-** | **+** | **-** | **+** | **-** |
| Evidence review: Addressing the social determinants of inequities in tobacco use (67) | **-** | **-** | **-** | **+** | **-** | **-** |
| The role of urban food policy in preventing diet-related non-communicable diseases in Cape Town & New York (9) | **+** | **+** | **+** | **+** | **-** | **-** |
| Social determinants and lifestyles: integrating environmental and public health perspectives(68) | **-** | **-** | **-** | **-** | **-** | **+** |
| Health in All Policies: a manual for local government(69) | **-** | **-** | **+** | **-** | **+** | **+** |
| Will the Meikirch Model, a New Framework for Health, Induce a Paradigm Shift in Healthcare?(12) | **-** | **-** | **-** | **-** | **-** | **+** |
| Keeping us well: How non-health charities address the social determinants of health(70) | **-** | **-** | **+** | **-** | **+** | **+** |
| Trade liberalization and social determinants of health: A state of the literature review(71) | **-** | **+** | **+** | **+** | **-** | **-** |
| Report of the Technical Consultation(72) | **-** | **-** | **+** | **-** | **-** | **-** |
