# Supplementary material for: Bringing the commercial determinants of health out of the shadows: a review of how the commercial determinants are represented in conceptual frameworks
Source: Eur J Public Health. 2020 Jan 18;30(4):660–4. doi: 10.1093/eurpub/ckz197 (PMC7445044; doi:10.1093/eurpub/ckz197)
Supplement: ckz197_Supplementary_Data [file ckz197_supplementary_data.zip › ejph-2019-07-om-0571-File003.docx]

**Supplementary material 2: Additional references (references to frameworks used in this study)**

34. Lalonde M. A new perspective on the health of canadians: a working document. Ontario: Government of Canada, 1974.

35. Department of Health and Social Services. Inequalities in health: Report of a research working group: s; 1980.

36. Evans R, Stoddart,GL., . Producing health, consuming healthcare. Social Science and Medicine 1990;31(12):1347-63.

37. Hamilton N, Bhatti,T.,. Population Health Promotion: An integrated model of population health and health promotion. Health Promotion Development Division, Canadian Government, 1996.

38. Berkman LF, Glass T, Brissette I, Seeman TE. From social integration to health: Durkheim in the new millennium. Soc Sci Med 2000;51(6):843-57.

39. Starfield B. Basic concepts in population health and health care. J Epidemiol Community Health 2001;55(7):452-4.

40. Centres for Disease Control and Prevention. The Social-Ecological Model: A Framework for Violence Prevention. 2002.

41. Schulz A, Northridge ME. Social determinants of health: implications for environmental health promotion. Health Educ Behav 2004;31(4):455-71.

42. Stahl T, Wismar M, Ollila E, Lahtinen E, Leppo K. Health in all policies: Prospects and Potentials. Finnish Ministry of Social Affairs and Health, 2006.

43. Etches V, Frank J, Di Ruggiero E, Manuel D. Measuring population health: a review of indicators. Annu Rev Public Health 2006;27:29-55.

44. Marmot M.G. WRG. Social Determinants of Health. Oxford: Oxford University Press; 2006.

45. Whitehead MD, G. Concepts and principles for tackling social inequities in health: Levelling up Part 1. University of Liverpool: WHO Collaborating Centre for Policy Research on Social Determinants of Health, 2006.

46. Whitehead M. A typology of actions to tackle social inequalities in health. J Epidemiol Community Health 2007;61(6):473-8.

47. Dyck M. Social Determinants of Metis Health. National Aboriginal Health Organization, 2008.

48. Hiatt RA, Breen N. The social determinants of cancer: a challenge for transdisciplinary science. Am J Prev Med 2008;35(2 Suppl):S141-50.

49. Fox AM, Meier BM. Health as freedom: addressing social determinants of global health inequities through the human right to development. Bioethics 2009;23(2):112-22.

50. Raphael D. Social Determinants of Health. 2nd Edition ed. Toronto, Ontario: Canadian Scholars' Press Inc; 2009.

51. Bambra C, Gibson M, Sowden A, Wright K, Whitehead M, Petticrew M. Tackling the wider social determinants of health and health inequalities: evidence from systematic reviews. J Epidemiol Community Health 2010;64(4):284-91.

52. Bozorgmehr K. Rethinking the 'global' in global health: a dialectic approach. Global Health 2010;6:19.

53. Mikkonen J, Raphael, D.,. Social Determinants of Health: The Canadian Facts. Toronto: York University School of Health Policy and Management, 2010.

54. Organisation WH. A Conceptual Framework for Action on the Social Determinants of Health. World Health Organisation, 2010.

55. Braveman P, Egerter S, Williams DR. The social determinants of health: coming of age. Annu Rev Public Health 2011;32:381-98.

56. National Research C, Institute of M. The National Academies Collection: Reports funded by National Institutes of Health. In: Woolf SH, Aron L, editors. US Health in International Perspective: Shorter Lives, Poorer Health. Washington (DC): National Academies Press (US) National Academy of Sciences.; 2013.

57. Krumeich A, Meershoek A. Health in global context; beyond the social determinants of health? Glob Health Action 2014;7:23506.

58. World Health Organization’s Commission on the Social Determinants of Health. Total Environment Assessment Model for Early Child Development. Geneva: World Health Organisation, 2014.

59. World Health Organisation. Health in All Policies: Helsinki statement. Framework for country action. World Health Organisation, 2014.

60. Friel S, Hattersley, L., Ford, L.,. Evidence review: Addressing the social determinants of inequities in healthy eating. Victorian Health Promotion Foundation, 2015.

61. Foundation VHP. Fair Foundations: The VicHealth framework for health equity. 2015.

62. Newman L, Javanparast, S, Baum, F., Hutchinson, C. Evidence review: Settings for addressing the social determinants of health inequities. Victorian Health Promotion Foundation, 2015.

63. Roche A, Kostadinov, V., Fischer, J, Nicholas, R. Evidence review: The social determinants of inequities in alcohol consumption and alcohol-related health outcomes. Victorian Health Promotion Foundation, 2015.

64. Moore T, McDonald, M., McHugh-Dillon, H. Evidence review: Early childhood development and the social determinants of health inequities. Victorian Health Promotion Foundation, 2015.

65. Ball K, Carver, A., Jackson, M, Downing, K. Evidence review: Addressing the social determinants of inequities in physical activity and related health outcomes. Victorian Health Promotion Foundation, 2015.

66. Barraket J, Mason, C., Friel, S. Evidence review: Social innovation for health equity promotion. Victorian Health Promotion Foundation, 2015.

67. Purcell K. Evidence review: Addressing the social determinants of inequities in tobacco use. Victorian Health Promotion Foundation, 2015.

68. Graham H, White PC. Social determinants and lifestyles: integrating environmental and public health perspectives. Public Health 2016;141:270-8.

69. Local Government Association. Health in All Policies: A manual for local government. 2016.

70. Boswell K, Joy I, Lamb, C. Keeping us well: How non-health charities address the social determinants of health. NPC, 2017.

71. McNamara C. Trade liberalization and social determinants of health: A state of the literature review. Soc Sci Med 2017;176:1-13.

72. WHO Independent High-level Commission on NCDs. Report of the Technical Consultation. Geneva: World Health Organisation, 2018.
